# Potential cure of Alzheimer's disease by reducing the level of Cdk5 using two drugs, each with a different modus operandi

**DOI:** 10.1177/25424823251335870

**Published:** 2025-04-15

**Authors:** Jeffrey Fessel

**Affiliations:** 1 Department of Medicine, University of California, San Francisco, San Francisco, CA, USA

**Keywords:** Alzheimer's disease, cellular biology, Cdk5, dementia, roscovitine, tamoxifen

## Abstract

There is need to understand the direct cause and, therefore, the appropriate treatment, of Alzheimer's disease (AD). A Google search was used to discern a) the primary cause of AD, and b) its treatment. Activation of Cdk5 is the primary cause for AD. Activation of Cdk5 may be decreased by using two drugs, roscovitine and tamoxifen, each having a different mechanism of action. Clinical trials should validate the efficacy and safety of using roscovitine and tamoxifen.

Abnormal thinking, as occurs in such disorders as Alzheimer's disease (AD), must largely depend upon neurotoxicity whether caused by genetical or environmental adversities or both. A major cause of neurotoxicity is active cyclin-dependent kinase 5 (Cdk5).^1^

Google Scholar was searched for articles relating to Cdk5 in AD, and methods of reducing its activation.

Abundant literature shows that prevention of neurotoxicity could be accomplished by preventing hyperactivation of Cdk5, whose levels are increased in the brains of patients with AD,^2^ and whose inhibition rescued the AD pathology seen in the brains of AD model mice.^3^ Active Cdk5 affects neural physiology, axonal growth, neurite outgrowth, synapse formation and neurotransmission, cognitive functions, and cell cycle re-entry. Normal Cdk5 activity is mainly caused by association with its primary activator p35. During neurotoxicity, p35 is cleaved by calpain to form p25, which then becomes a major, hyper-activator of Cdk5. P25 levels and, thus, also active Cdk5, were elevated in AD brain; and overexpression of p25 in a transgenic mouse resulted in the formation of phosphorylated tau, neurofibrillary tangles and cognitive deficits, that are all present in human AD.^4^ Thus, inhibition of Cdk5 activity should have a high presumptive likelihood of reversing AD.

An approach using inhibitors of Cdk5, with each agent using a different mechanism for its inhibiting effect, might, therefore, provide a reasonable likelihood of reversing AD. Two such drugs, each having FDA approval, are presented here. The first drug is roscovitine, that crosses the blood-brain barrier and affects the ATP binding site of Cdk5 (Lys33); roscovitine decreased Cdk5 activity by 87.2% after cerebral ischemia.^5^

The second drug is tamoxifen, that binds to the p35 or p25 activators of Cdk5 and reduced Ckd5 activity by 84.5%.^6^ The side effects of tamoxifen require discussion. In a literature review, Wibowo et al. found 14 randomized, controlled studies (RCTs) with 312 men, and 39 non-randomized studies with 150 men.^7^ Tamoxifen, in the RCTs in men with prostate cancer, gynecomastia, or mastalgia, was administered at 10 or 20 mgs daily for 6–36 months; and in the non-randomized studies of men with prostate cancer, breast cancer, or idiopathic gynecomastia, tamoxifen was administered at 10–160 mgs/day for 2–16 months. Adverse events (AE) in those participating in the RCTs included 72 (23.1%) with gynecomastia, 72 (23.1%) with mastalgia, 27 (8.7%) with either hot flashes or vasodilatation, 23 (7.4%) with either diarrhea or constipation, 17 (5.4%) with fatigue, and 16 (5.1%) with various infections particularly upper respiratory, and fewer with a variety of other AE; in the 454 men with breast cancer not participating in the RCTs, 44 (9.7%) experienced decreased libido, 33 (7.3%) had hot flashes, and 31 (6.9%) had fatigue. The small difference in hot flashes is probably because the higher rate included subjects with vasodilatation, that was not assessed in the other group. Tamoxifen being an anti-estrogen is a presumptive mechanism for the hot flashes.

For 42 days, 57 men received either the anti-androgen bicalutamide plus placebo or bicalutamide plus the anti-estrogen tamoxifen.^8^ Vasodilation occurred in 5.6% of those taking bicalutamide plus placebo and in 14.7% of those taking bicalutamide plus tamoxifen, and decreased libido in 2.8% versus 5.9% respectively, which indicates the increases in vasodilation or decreases in libido that are attributable to tamoxifen taken alone, as being 75.5% and 52.5% respectively. In a double blind, cross-over study of either placebo or tamoxifen 10 mgs bid, seven of ten patients experienced a decrease in the size of gynecomastia (*p* < 0.005), presumably caused by the anti-estrogenicity of tamoxifen.^9^ In 150 men with prostate cancer, treated with tamoxifen, loss of weight occurred in only 4 (2.7%); the most common side effects being nausea in 30 (20%) and altered gait in 20 (13.3%), with decreased libido being seen in only one subject which, again, is probably because tamoxifen is anti-estrogen but not anti-androgen.^7^

Thus, the concurrent, dual administration of roscovitine, and tamoxifen might reduce Cdk5 to a level where it is effectively, no longer active and, therefore, be a presumptive cure of AD. It should be noted that approximately 20% of men given tamoxifen might experience weight gain, sexual problems, or hot flashes. For this reason, initial trials should be performed in women with AD. Because of their possible toxicities, each drug in the required, initial treatment trial should be administered to both men and women at half the usual dosage; if no adverse events occur, then a second trial, also in both sexes, would use full dosages. Although this author has published several other suggestions for reversing AD, the approach presented here would seem to provide the highest, presumptive likelihood of success.
